# Intranasal Immunization with a Recombinant Adenovirus Encoding Multi-Stage Antigens of *Mycobacterium tuberculosis* Preferentially Elicited CD8^+^ T Cell Immunity and Conferred a Superior Protection in the Lungs of Mice than Bacillus Calmette–Guerin

**DOI:** 10.3390/vaccines12091022

**Published:** 2024-09-06

**Authors:** Limei Wang, Jian Kang, Hong Jiang

**Affiliations:** 1 Bacteriology Laboratory, Department of Microbiology and Pathogenic Biology, School of Basic Medicine, Air Force Medical University, Xi’an 710032, China; limwang@126.com (L.W.); kangjianhy88@126.com (J.K.); 2 Center for Diagnosis and Treatment of Infectious Diseases, Second Affiliated Hospital, Air Force Medical University, Xi’an 710038, China

**Keywords:** tuberculosis, *Mycobacterium tuberculosis*, vaccine, recombinant adenovirus, intranasal immunization, multi-stage antigens

## Abstract

The development of a tuberculosis (TB) vaccine is imperative. Employing multi-stage *Mycobacterium tuberculosis* (Mtb) antigens as targeted antigens represents a critical strategy in establishing an effective novel TB vaccine. In this investigation, we evaluated the immunogenicity and protective efficacy of a recombinant adenovirus vaccine expressing two fusion proteins, Ag85B-ESAT6 (AE) and Rv2031c-Rv2626c (R2), derived from multi-stage antigens of Mtb via intranasal administration in mice. Intranasal delivery of Ad-AE-R2 induced both long-lasting mucosal and systemic immunities, with a preferential elicitation of CD8^+^ T cell immunity demonstrated by the accumulation and retention of CD8^+^ T cells in BALF, lung, and spleen, as well as the generation of CD8^+^ TRM cells in BALF and lung tissues. Compared to subcutaneous immunization with Bacillus Calmette-Guerin (BCG), Ad-AE-R2 provided superior protection against high-dose intratracheal BCG challenge, specifically within the lungs of mice. Our findings support the notion that empowering T cells within the respiratory mucosa is crucial for TB vaccine development while highlighting targeting CD8^+^ T cell immunity as an effective strategy for optimizing TB vaccines and emphasizing that eliciting systemic memory immunity is also vital for the successful development of a TB mucosal vaccine. Furthermore, our results demonstrate that the BCG challenge serves as a convenient and efficient method to evaluate candidate vaccine efficacy.

## 1. Introduction

Tuberculosis (TB) remains the leading cause of death worldwide caused by a single infectious agent. It is estimated that approximately 1/4 of the global population is infected with *Mycobacterium tuberculosis* (Mtb), and 5~10% of these individuals will develop active TB at some point in their lifetime [1]. Bacillus Calmette–Guerin (BCG) is presently the only available vaccine against TB and is administered intradermally shortly after birth. BCG offers protection against childhood forms of TB, such as TB meningitis and miliary TB. However, its immune effectiveness diminishes within 10 to 15 years, rendering it ineffective in preventing pulmonary TB in adolescents and adults [2,3]. Therefore, there is an urgent need for a new and more potent vaccine against TB.

Adenovirus (Ad) vectors are considered one of the most promising vaccine platforms due to their potential advantages, including type I immune adjuvant properties, large transgene capacity, excellent safety profile in humans, self-limited but prolonged high levels of antigen release, and suitability for both parenteral and intranasal delivery [4]. Ad vector-based vaccines have been developed for various pathogens, such as HIV, HCV, influenza, Ebola, and Zika virus infections [5]. In response to the COVID-19 pandemic, Ad vector-based vaccines have also demonstrated successful protection against COVID-19 in human trials [6]. Jeyanathan M. et al. [7] developed a recombinant Ad vector-based TB vaccine called AdHu5Ag85A, which expresses Mtb antigen Ag85A. The safety and immunogenicity of AdHu5Ag85A have been evaluated through phase Ib clinical trials using the aerosolized inhalation vaccination route [8]. The results have shown that the aerosolized inhalation delivery of AdHu5Ag85A induces safe and superior respiratory mucosal immunity, suggesting further development potential against TB [8].

Mtb, a facultative intracellular pathogen, is capable of residing within macrophage cells and causing latent infection. In this case, Mtb survives in the macrophage in a dormant state [9]. The dormant Mtb bacilli express a group of dormancy-related proteins, most of which are barely transcribed and expressed in the replicating Mtb bacilli during the acute stage of infection [9]. The expression of different antigens at various stages of infection represents one of the immune-evasive strategies employed by Mtb [10]. Accumulating studies on novel TB vaccines have shown that using multi-stage antigens comprising antigens from both replicating and dormant Mtb bacilli is a crucial strategy aimed at establishing comprehensive immunity capable of protecting against active symptomatic and latent asymptomatic forms of Mtb infection [11,12,13]. Although AdHu5Ag85A has shown promising results in Phase Ib clinical trials [8], it only targets a single-stage Mtb antigen, Ag85A, which is predominantly produced during the acute stage of infection and cannot effectively target dormant Mtb bacilli for their eradication.

Therefore, to develop a more comprehensive TB vaccine that is immunogenic and protective, we generated a recombinant Ad, Ad-AE-R2. This recombinant Ad expressed Mtb multi-stage antigens of Ag85B, ESAT6 from replicating bacteria, and Rv2031c, Rv2626c from dormant bacteria as two fusion proteins (Ag85B-ESAT6 [AE] and Rv2031c-Rv2626c [R2]) under the control of separate promoters CMV and human EF1α. Our findings demonstrate that intranasal immunization with Ad-AE-R2 can effectively stimulate durable mucosal immunity and systemic memory immunity while preferentially inducing CD8^+^ T cell-mediated immune responses. Compared to subcutaneous immunization with BCG, Ad-AE-R2 provides superior protection in the lung and comparable protection in the spleen of mice against high-dose intratracheal BCG challenge. These results support the notion that enhancing T-cell responses in the respiratory mucosa is crucial for TB vaccine development. Furthermore, targeting CD8^+^ T cell-mediated immunity represents an effective strategy for optimizing TB vaccines while eliciting systemic memory immunity is also important for the successful development of a mucosal TB vaccine. Additionally, our study demonstrates that the BCG challenge serves as a convenient and efficient method for evaluating vaccine candidate efficacy.

## 2. Materials and Methods

### 2.1. Mice

Six- to eight-week-old wild-type female C57BL/6 mice were purchased from the Laboratory Animal Center, Air Force Medical University (Xi’an, China). Mice were housed in a specific pathogen-free, level B facility. All of the experiments were performed under institutional guidelines from the Animal Research and Ethics Board.

### 2.2. Cells and Mycobacteria

HEK293T cells (catalog no. GDC187, CCTCC) were maintained in DMEM (HyClone, Logan, UT, USA) and supplemented with 10% FBS (Gibco, Vacaville, CA, USA), 100 U/mL penicillin, and 0.1 mg/mL streptomycin (HyClone, UT, USA). BCG-Beijing, for vaccination and challenge, was cultivated to the mid-log phase in 7H9 broth (Difico, Detroit, MI, USA) supplemented with 10% OADC and 0.5% Tween 80, then frozen in aliquots at −80 °C. Before use, the bacteria were washed in PBS and sonicated briefly. The number of bacteria was enumerated by plating on 7H10 agar supplemented with 10% OADC and 0.5% Tween 80.

### 2.3. Recombinant Adenovirus Ad-AE-R2

The production of a recombinant adenovirus Ad-AE-R2 has been described previously (Figure S1A) [14]. Briefly, HEK293T cells were transfected with *Pac*Ⅰ predigested recombinant adenovirus vector pAdeasy-CMV-AE-EF1α-R2 (RRID: Addgene_194928). Viruses were purified via plaque purification and propagated in HEK293T cells. Then, the proliferated viruses were obtained using standard CsCl gradient centrifugation and formulated in phosphate-buffered saline–5% sucrose after dialysis [14].

### 2.4. Recombinant AE and R2 Fusion Proteins

The recombinant AE and R2 fusion proteins used were produced and purified following established methods [15]. DNA constructs of Ag85B-ESAT6 (AE) or Rv2031c-Rv2626c (R2) for expression in *E. coli* were inserted into the prokaryotic expression vector pProEX-Htb (RRID:Addgene_1176). The recombinant vectors were transformed into *E. coli* DH5α, and protein expression was induced with 1 mM isopropyl ß-d-1-thiogalactopyranoside in 2 L cultures. AE was purified from inclusion bodies, and R2 was purified from supernatants via Ni-NTA chromatography.

## 3. BCG Subcutaneous Immunization and Intranasal Immunization of Ad-AE-R2

**Figure 1:**
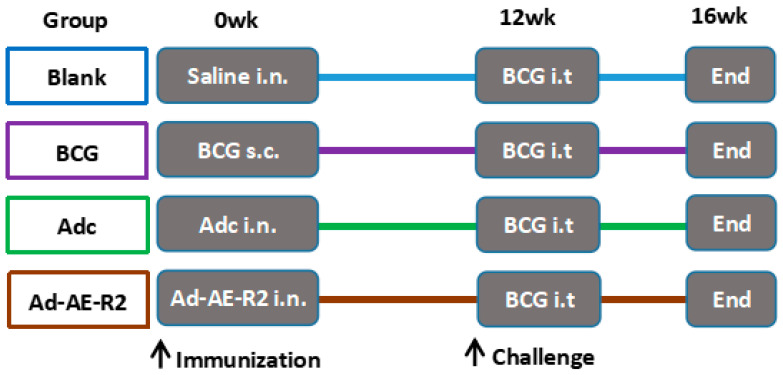
Study design: A total of 72 6~8 wks C57BL/6 mice were randomly divided into four groups: blank group, unvaccinated normal control; BCG group, vaccinated with BCG-Beijing subcutaneously at the back of the neck with a dose of 2 × 10^6^ CFU/50 µL PBS per mouse; Adc group, immunized intranasally with a replication-deficient adenovirus type 5 empty vector, 10^8^ PFU/25 µL PBS per mouse; and Ad-AE-R2 group, immunized intranasally with recombinant adenovirus expressing Ag85B-ESAT6 (AE) and Rv2031c-Rv2626c (R2) fusion proteins, 10^8^ PFU/25 µL PBS per mouse. Then, 12 weeks after immunization, 6 mice in each group were challenged with 10^8^ CFU BCG-Beijing through intratracheal and monitored for 4 weeks post challenge (study endpoint).

Figure 1 illustrates the experimental schedule starting with week zero. The mice were randomly divided into four groups: the blank group, the empty Ad vector control (Adc) group, the BCG group, and the Ad-AE-R2 group. There were 18 mice per group. Mice in the blank group received PBS via the intranasal route as a control. Mice in the Adc group and Ad-AE-R2 group were immunized via the intranasal route with Adc or Ad-AE-R2, 10^8^ plaque-forming units (PFUs) in 25 µL PBS per mouse. Mice in the BCG group were vaccinated subcutaneously at the back of the neck with 2 × 10^6^ colony-forming units (CFUs) BCG-Beijing in 50 μL PBS per mouse.

### 3.1. Immunogenicity Studies in Mice

Twelve weeks after immunization, 10 mice in each group were euthanized via exsanguination for immunogenicity studies. Bronchoalveolar lavage fluid (BALF), peripheral blood, spleens, and lungs were collected for analysis.

### 3.2. Secretory IgA (sIgA) Measurement in BALF

The collection of BALF followed a previously described method [16]. The supernatant of BALF was either immediately used to measure sIgA or stored at −20 °C. An Enzyme-linked immunosorbent assay (ELISA) kit (Cloud-Clone, Wuhan, China) was employed to determine the levels of sIgA, as per the manufacturer’s instructions.

### 3.3. Identification of Antibodies Specific to Antigens in Serum

Individual mice were subjected to peripheral blood sample collection. The levels of serum antigen-specific immunoglobulin G (IgG), IgG1, and IgG2a were assessed using ELISA kits in accordance with the instructions provided by the manufacturer (Mabtech, Stockholm, Sweden). The purified Mtb fusion protein AE (10 μg/mL), R2 (10 μg/mL), AE + R2 (containing 5 μg/mL AE and 5 μg/mL R2) or the whole-cell lysates (WCLs) of BCG (10 μg/mL) were used as antigens to coat the plates, respectively.

### 3.4. Preparation of Single-Cell Suspensions

Single-cell suspensions were prepared from BALF, lungs, and spleens. Cells from BALF and spleen tissues were isolated as described previously [6,17]. Lungs were cut into small pieces, and then digested with collagenase type Ⅰ (150 U/mL, Sangon, Shanghai, China) at 37 °C, 5% CO_2_ for one hour. The lung homogenate and spleens were minced through 70 μm cell strainers using a 5 mL syringe plunger. The cells were collected via centrifugation. The cell pellet was suspended in 4 mL of 40% Percoll and layered onto 4 mL of 70% Percoll, then centrifuged at 2000 rpm at 4 °C for 25 min. The isolated cells were washed twice in cold DPBS followed by 5 min centrifugation at 1000 rpm. Finally, all cells were resuspended in RPMI medium containing 10% fetal calf serum (FCS). Cell numbers were determined using trypan blue and adjusted to 1 × 10^7^ cells/mL [18].

### 3.5. Lymphocyte Proliferation Assay

An amount of 5 × 10^5^ splenocytes were placed in a single well of a 96-well plate and cultured in 10% FCS RPMI 1640 medium with or without each of the following antigens: AE (10 μg/mL), R2 (10 μg/mL), AE + R2 (5 μg/mL AE and 5 μg/mL R2), or BCG WCL (10 μg/mL) at 37 °C, 5% CO_2_ for 72 h. Cell proliferation was examined using the Cell Counting Kit-8 (CCK8) viability assay according to the manufacturer’s protocol (Dojindo, KMJ, Kumamoto, Japan). The proliferative responses were quantified using a stimulation index (SI), which was determined using the following formula: SI = mean of OD value of the antigen-stimulated cells/mean of OD value of the control cells. Each experiment was conducted in triplicate.

### 3.6. Cell Surface Staining

Cell surface staining and flow cytometry were performed as previously described [19,20]. Briefly, 100 μL of 10^7^ cells/mL single-cell suspension from each BALF, lung, and spleen sample was added to flow cytometry tubes and incubated with a LIVE/DEAD Yellow Stain Kit (Invitrogen^TM^ Thermo Fisher Scientific, Waltham, MA, USA] at 4 °C for 30 min. Cells were washed and blocked with anti-CD16/CD32 (93, Biolegend, San Diego, CA, USA) in 0.5% FCS-PBS at 4 °C for 15 min and then labeled with fluorochrome-labeled mAbs at 4 °C for 25 min. The surface identification of T cells was performed with antibodies CD3 eFluor 450 (17A2, eBioscience, Frankfurt am Main, Germany), CD4 FITC (GK4.5, Biolegend), CD8 PerCP (53–6.7, Biolegend), CD44 Alexa Fluor (IM7, Biolegend, San Diego, CA, USA), CD69 PE-Cy7 (H1.2F3, Biolegend, San Diego, CA, USA), CD103 PE (2E7, Biolegend, San Diego, CA, USA), and CD62L APC-Cy7 (MEL-14, Biolegend, San Diego, CA, USA). Stained cells were processed according to Biolegend instructions for flow cytometry and run on a BD FACSCanto flow cytometer. The data were analyzed using FlowJo^TM^ software (version 10.1; Tree Star, Ashland, OR, USA).

### 3.7. Intracellular Cytokine Staining and Flow Cytometry Analysis

The cell suspension obtained via the above procedures was cultured in a 24-well plate at a concentration of 10^7^ cells/mL for lung and spleens. The cells were cultured with each of the following antigens: AE (10 μg/mL) or R2 (10 μg/mL) or AE + R2 (5 μg/mL AE and 5 μg/mL R2) or BCG WCL (10 μg/mL) at 37 °C for 18 h. For intracellular cytokine staining (ICS), brefeldin A (5 μg/mL, Sigma-Aldrich, St. Louis, MO, USA) was added for an additional 6 h after stimulation, and the cells were collected using 0.5% FCS-PBS, stained with a LIVE/DEAD™ Yellow Stain Kit (Invitrogen^TM^ Thermo Fisher Scientific, MA, USA) at 4 °C for 20 min, sheltered from light and washed with 0.5% FCS-PBS. Surface markers were labeled with antibodies CD16/CD32 (93, Biolegend, San Diego, CA, USA), CD3 eFluor 450 (17A2, eBioscience, Frankfurt am Main, Germany ), CD4 APC-Cy7 (GK1.5, Biolegend, San Diego, CA, USA), and CD8 PerCP (53–6.7, Biolegend, San Diego, CA, USA) at 4 °C for 20 min. Then, the cells were fixed and permeabilized with reagent following the manufacturer’s protocols from the Biolegend intracellular fixation/permeabilization set. The cells were stained with intracellular cytokines of IFN-γ, IL-2, IL-17A, and TNF-α at 4 °C for 20 min, and the samples were washed and resuspended with 0.5% FCS-PBS. The following antibodies were used for ICS: IFN-γ FITC (XMG1.2, Biolegend, San Diego, CA, USA), IL-2 APC (JES6-5H4, Biolegend, San Diego, CA, USA), IL-17A PE (TC11-18H10.1, Biolegend, San Diego, CA, USA), and TNF-α BV510 (MP6-XT22, Biolegend, San Diego, CA, USA).

### 3.8. Intratracheal BCG Challenge

Twelve weeks after immunization, 6 mice in each group were challenged intratracheally with 10^8^ CFU BCG-Beijing. Before use, the stocked BCG bacteria were washed with PBS containing 0.05% Tween 80 and passed through a 27-gauge needle ten times to disperse clumps. For the intratracheal BCG challenge, the mice were anesthetized by administering sodium pentobarbital (50 mg/kg) intraperitoneally prior to being positioned on a mouse intubation platform inclined at a 45° angle. The mice were immobilized by securing a nylon wire under their front incisors and placing a lateral barrier across their chest. Each mouse’s mouth was gently opened, and forceps were used to position the tongue to one side. To accurately locate the trachea and vocal cords, a fiber optic light source was employed prior to inserting a 40 mm plastic sheath into the trachea. The plastic intubation sheath was used to guide the blunt needle portion of a microinjector to reach the bifurcation of the trachea, where the inoculum containing 100 µL PBS with 10^8^ CFU BCG was delivered [21].

### 3.9. Enumeration of Bacterial Burden in Organs

To assess the effectiveness of the vaccine, the number of BCG colony-forming units (CFUs) was counted in the lungs and spleens of mice four weeks after exposure. The lung and spleen tissues were homogenized in 5 mL of PBS. The tissue homogenates were diluted in a series, plated onto Middlebrook 7H10 plates, and incubated at 37 °C and 5% CO_2_ for 3~4 weeks. CFU data were transformed logarithmically before analysis.

### 3.10. Statistics

GraphPad Prism 9.3 was used for statistical analysis and graph preparation. Unless specified otherwise, datasets were subjected to one or two-way ANOVA with Tukey’s multiple comparisons test performed on the datasets. All of the error bars represent the standard error of the mean, while midlines represent the group means. A significance level of *p* < 0.05 was applied to all experiments.

## 4. Results

### 4.1. Characteristics of the Recombinant Adenovirus Ad-AE-R2 Expressing Mtb Multi-Stage Antigens

The recombinant adenovirus Ad-AE-R2 was designed to encode multi-stage antigens of Mtb in two fusion proteins, Ag85B-ESAT6 (AE) and Rv2031c-Rv2626c (R2), as Mtb expresses different antigens during its replicating stage and dormancy stage. The genes of Ag85B and ESAT6, which are immunodominant antigens from replicating bacteria of Mtb, were linked by a hydrophobic linker (GlySer3)4, and the fusion gene was controlled by a human CMV promoter. Similarly, the genes of Rv2031c and Rv2626c, immunodominant antigens from dormant bacteria of Mtb, were also linked by the hydrophobic linker (GlySer3)4, with the fusion gene under the control of a human EF1α promoter (Figure S1A). To assess gene cassette expression in eukaryotic cells infected with the recombinant adenovirus Ad-AE-R2, qRT-PCR and indirect immunofluorescence assay (IFA) were performed using monoclonal antibodies against Ag85B, ESAT6, Rv2031c, and Rv2626c antigens, respectively. The results demonstrated successful transcription and the expression of both AE and R2 fusion proteins in infected eukaryotic cells (Figure S1B,C) [14].

### 4.2. Intranasal Immunization with Ad-AE-R2 Preferentially Induces the Accumulation of CD8^+^ T Cells in the Airway and Facilitates the Generation of CD8^+^ TRM Cells

**Figure 2:**
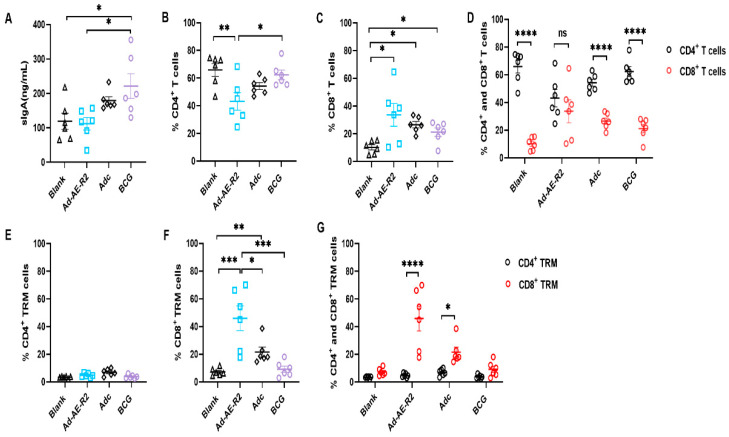
Immunological characteristics in BALF of immunized mice (n = 6 per group). (**A**) sIgA contents; (**B**) percentages of CD4^+^ T cells; (**C**) percentages of CD8^+^ T cells; (**D**) the comparison of percentages of CD4^+^ and CD8^+^ T cells; (**E**) percentages of CD4^+^ TRM cells; (**F**) percentages of CD8^+^ TRM cells; (**G**) the comparison of percentages of CD4^+^ and CD8^+^ TRM cells. *, *p* < 0.05; **, *p* < 0.01; ***, *p* < 0.001; ****, *p* < 0.0001; ns, no statistical significance.

The aim of TB mucosal vaccines is to induce antigen-specific mucosal immunity in the respiratory tract [22]. Therefore, we intranasally immunized mice with Ad-AE-R2 and assessed the nature of mucosal immunity in immunized mice 12 weeks after immunization to allow for the contraction of adaptive immune responses. SIgA is the predominant antibody isotype produced in mucosal tissues [23]. We evaluated the sIgA content in the BALF of mice using ELISA. The results showed that there was no significant difference in sIgA content between the Ad-AE-R2 group and the blank group, while it was higher in the BCG group compared to both the blank and Ad-AE-R2 groups (Figure 2A).

T cells present in the airway lumen but not in the peripheral tissue compartments play a critical role in TB protection [22]. Subsequently, we analyzed CD4^+^ and CD8^+^ T cells in the BALF of mice using flow cytometry. We observed that intranasal immunization with Ad-AE-R2 significantly decreased the percentage of CD4^+^ T cells compared to the blank group while increasing the percentage of CD8^+^ T cells, resulting in comparable percentages of CD4^+^ and CD8^+^ T cells in the BALF of mice. Both BCG and Adc did not alter the percentage of CD4^+^ T cells in the BALF of mice; however, they also increased the percentage of CD8^+^ T cells, although not as significantly as observed in the Ad-AE-R2 group. In Adc and BCG groups, CD4^+^ T cells were still predominant among total lymphocytes in the BALF of mice, as in the blank group (Figure 2B–D).

TRM cells, a subset of lymphocytes residing in localized tissue compartments and lacking recirculation into the bloodstream [24], serve as the primary defense against Mtb infection within local tissues and exhibit rapid responsiveness to potential Mtb infection [20]. Therefore, we employed flow cytometry to detect TRM cells in the BALF of mice (Figure S2). The results revealed that compared to the blank group, there was no significant change in the percentage of CD4^+^ TRM cells observed in Ad-AE-R2, Adc, and BCG groups (Figure 2E). However, there was a notable increase in the percentage of CD8^+^ TRM cells observed in both Ad-AE-R2 and Adc groups, with a more pronounced increase seen in the Ad-AE-R2 group compared to the Adc group (Figure 2F). Consequently, CD8^+^ TRM cells predominated within the BALF of mice belonging to both Ad-AE-R2 and Adc groups. In contrast, no significant difference was found between CD4^+^ and CD8^+^ TRM cell percentages within the BALF of mice belonging to either the blank or BCG groups (Figure 2G).

### 4.3. Intranasal Immunization with Ad-AE-R2 Selectively Amplifies the Proportion of CD8^+^ T Cells in the Lung of Immunized Mice While Also Inducing a Higher Proportion of CD8^+^ T Cells That Produce Cytokines Compared to BCG

**Figure 3:**
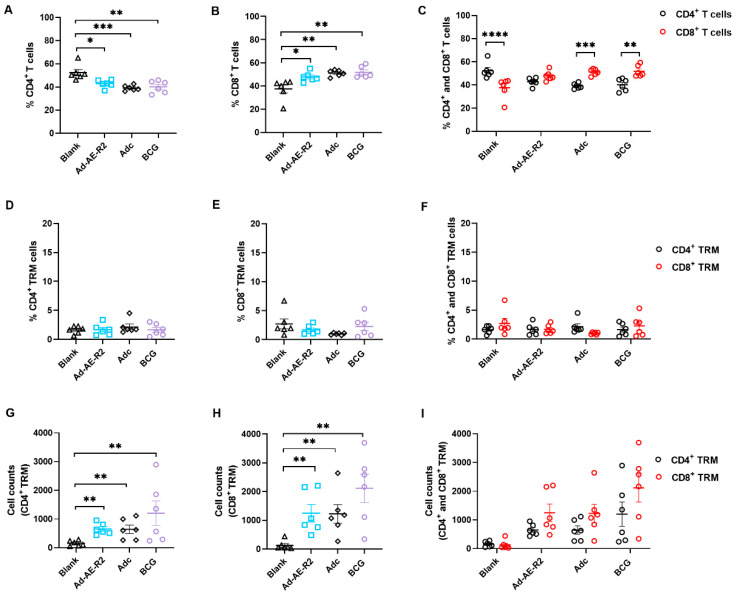
Phenotype of T cells in the lung of immunized mice (n = 6 per group). (**A**) Percentages of CD4^+^ T cells; (**B**) percentages of CD8^+^ T cells; (**C**) the comparison of percentages of CD4^+^ and CD8^+^ T cells; (**D**) percentages of CD4^+^ TRM cells; (**E**) percentages of CD8^+^ TRM cells; (**F**) the comparison of percentages of CD4^+^ and CD8^+^ TRM cells; (**G**) numbers of CD4^+^ TRM cells; (**H**) numbers of CD8^+^ TRM cells; (**I**) the comparison of numbers of CD4^+^ and CD8^+^ TRM cells. *, *p* < 0.05; **, *p* < 0.01; ***, *p* < 0.001; ****, *p* < 0.0001.

TB primarily affects the lungs, making local immunity in the lungs crucial for protective immunity against TB [25]. To examine T cell immunity in the lung of immunized mice, we initially analyzed the proportions of CD4^+^ and CD8^+^ T cells using flow cytometry. The results showed that in the Ad-AE-R2, Adc, and BCG groups, the percentages of CD4^+^ T cells decreased, while the percentages of CD8^+^ T cells increased in the lung compared to the blank group. There were no differences in the percentages of CD4^+^ and CD8^+^ T cells among Ad-AE-R2, Adc, and BCG groups (Figure 3A,B). Notably, while both CD4^+^ and CD8^+^ T cells were comparable in percentage within the lung of mice from the Ad-AE-R2 group, predominance was observed for CD8^+^ T cells in both Adc and BCG groups as well as for CD4^+^ T cells in the blank group (Figure 3C).

Subsequently, we assessed the presence of CD4^+^ and CD8^+^ TRM cells by analyzing their proportions within each group. Interestingly, there were no differences in either CD4^+^ and CD8^+^ TRM cell proportions among the groups (Figure 3D, E). Furthermore, comparable percentages of CD4^+^ and CD8^+^ TRM cells were found in all groups (Figure 3F). However, when we specifically quantified TRM cell numbers within each group, it was evident that Ad-AE-R2, Adc, and BCG immunized mice had significantly higher numbers of both CD4^+^ and CD8^+^ TRM cells compared to the blank group. Additionally, there were more CD4^+^ and CD8^+^ TRM cells in the BCG group than in other groups, although there were no statistical differences (Figure 3G, H). Nevertheless, the numbers of CD4^+^ and CD8^+^ TRM cells were similar within each group (Figure 3I).

**Figure 4:**
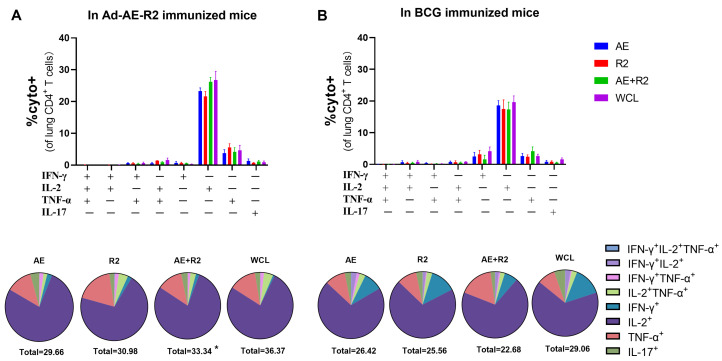
The percentages of CD4^+^ T cells secreting cytokines of IFN-γ, IL-2, TNF-α, or IL-17 in the lungs of mice in Ad-AE-R2 group and BCG group in response to stimulations with AE, R2, AE + R2, or BCG WCL proteins in vitro. (**A**) in Ad-AE-R2 immunized mice (n = 6); (**B**) in BCG immunized mice (n = 6). *, *p* < 0.05.

Th1 and Th17 cell-mediated immunity has been associated with immune protection against TB [26]. Therefore, we evaluated TNF-α, IFN-γ, IL-2, and IL-17-producing T cells upon ex vivo stimulations with AE, R2, AE + R2, and BCG WCL via flow cytometry in the lungs of mice from the Ad-AE-R2 group and BCG group. Our results revealed a similar cytokine profile among the responding cells in both groups. Notably, IL-2-producing cells constituted the majority of cytokine-producing CD4^+^ T cells in the lungs of mice from both groups. In the Ad-AE-R2 group, TNF-α-producing cells represented the second largest proportion of cytokine-producing CD4^+^ T cells, followed by IFN-γ-producing CD4^+^ T cells and IL-17-producing CD4^+^ T cells. Conversely, in the BCG group, TNF-α and IFN-γ single cytokine-producing CD4^+^ T cells accounted for the second-largest proportion of cytokine production, followed by IL-17-producing CD4^+^ T cells. Overall, only a statistical difference was observed upon stimulation with AE + R2; there was a greater proportion of cytokine-producing CD4^+^ T cells in the Ad-AE-R2 group compared to that in the BCG group (Figure 4).

**Figure 5:**
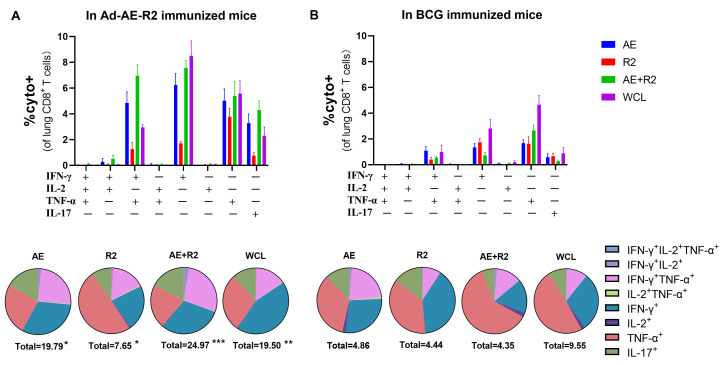
The percentages of CD8^+^ T cells secreting cytokines of IFN-γ, IL-2, TNF-α or IL-17 in the lung of mice in Ad-AE-R2 group and BCG group in response to stimulations with AE, R2, AE + R2,or BCG WCL proteins in vitro. (**A**) in Ad-AE-R2-immunized mice (n = 6); (**B**) in BCG-immunized mice (n = 6). *, *p* < 0.05; **, *p* < 0.01; ***, *p* < 0.001, comparison between Ad-AE-R2 group and BCG group.

The cytokine profile in CD8^+^ T cells was found to be similar between the Ad-AE-R2 group and the BCG group, regardless of whether the T cells were stimulated with AE, R2, AE + R2, or BCG WCL. Both groups exhibited comparable proportions of IFN-γ, TNF-α, and IL-17 single cytokine-producing CD8^+^ T cells; however, there was a lack of IL-2-producing CD8^+^ T cells in both groups. Additionally, both groups showed a proportion of bi-functional cytokine-producing CD8+ T cells producing IFN-γ and TNF-α. Notably, the Ad-AE-R2 group had a higher proportion of each type of cytokine-producing CD8^+^ T cell compared to the BCG group. Finally, irrespective of stimulation with AE, R2, AE + R2, or BCG WCL, the total proportion of cytokine-producing CD8^+^ T cells was significantly greater in the Ad-AE-R2 group than in the BCG group (Figure 5).

### 4.4. Intranasal Immunization with Ad-AE-R2 Selectively Enhances the Proportions of CD8^+^ T Cells and Induces the Generation of Tem and Tcm Cells in the Spleen of Mice

**Figure 6:**
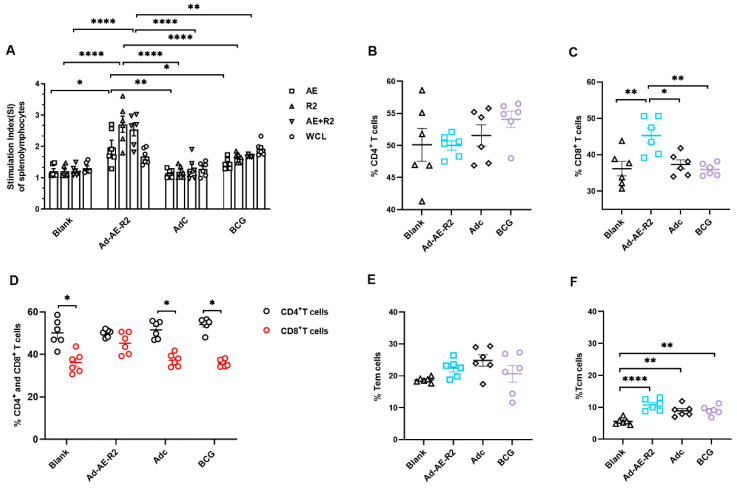
Antigen-specific proliferation and phenotype of T cells in the spleen of mice (n = 6 per group). (**A**) the stimulation index (SI) values of splenolymphocytes stimulated with AE, R2, AE + R2, or BCG WCL proteins in vitro. (**B**) percentages of CD4^+^ T cells; (**C**) percentages of CD8^+^ T cells. (**D**) the comparison of percentages of CD4^+^ and CD8^+^ T cells. (**E**) percentages of Tem cells. (**F**) percentages of Tcm cells. *, *p* < 0.05; **, *p* < 0.01; ****, *p* < 0.0001.

To evaluate T cell responses in the spleen of immunized mice, we assessed the proliferative capacity of antigen-specific lymphocytes in splenolymphocytes upon ex vivo stimulation with AE, R2, AE + R2, or BCG WCL. The results demonstrated that the stimulation index (SI) values were higher in the Ad-AE-R2 group compared to the blank, Adc, and BCG groups when splenolymphocytes were stimulated with AE, R2, or AE + R2 proteins. However, there was no difference in SI values between the Adc and BCG groups compared to the blank group, regardless of which protein was used for stimulation (Figure 6A).

Next, we aimed to investigate whether intranasal immunization with Ad-AE-R2 could enhance CD8^+^ T cell immune responses in the spleen. We analyzed the percentages of CD4^+^ and CD8^+^ T cells in the spleen of immunized mice. The results revealed that BCG preferentially increased the percentage of CD4^+^ T cells while Ad-AE-R2 preferentially increased the percentage of CD8^+^ T cells compared to the blank group (Figure 6B, C). There were no significant changes observed in CD4^+^ and CD8^+^ T cell percentages between the Adc group and the blank group. Thus, CD4^+^ T cells predominated in the spleens of mice in the blank, Adc, and BCG groups, whereas comparable percentages of CD4^+^ and CD8^+^ T cells were observed in the Ad-AE-R2 group (Figure 6D).

Immunological memory is a crucial characteristic of adaptive immune response, playing a vital role in conferring long-term specific immunity against pathogens and serving as a critical factor in vaccine exploration [27]. Subsequently, we examined the presence of Tem and Tcm cells in the spleen of mice. The results showed that the percentages of Tem and Tcm cells in Ad-AE-R2, Adc, and BCG groups were all increased compared to the blank group; however, there was no statistically significant difference in the percentage of Tem cells among the groups (Figure 6E). Notably, the percentage of Tcm cells in the Ad-AE-R2 group was significantly higher than that in both Adc and BCG groups, although statistical significance was not reached (Figure 6F).

**Figure 7:**
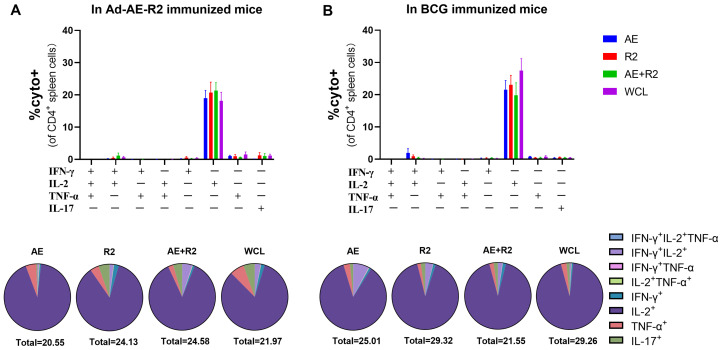
The percentages of CD4^+^ T cells secreting cytokines of IFN-γ, IL-2, TNF-α or IL-17 in the spleen of mice in Ad-AE-R2 group and BCG group in response to stimulations with AE, R2, AE + R2, or BCG WCL proteins in vitro. (**A**) In Ad-AE-R2-immunized mice (n = 6); (**B**) in BCG-immunized mice (n = 6).

**Figure 8:**
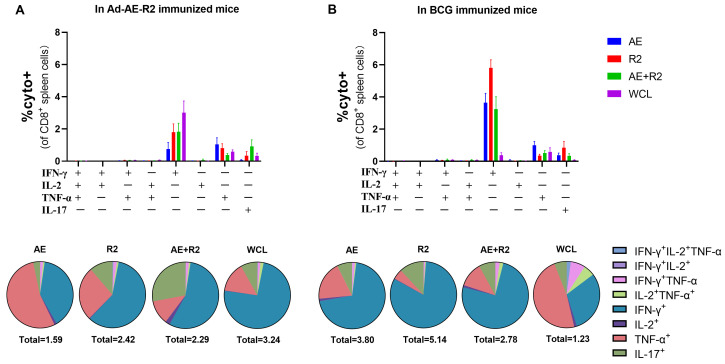
The percentages of CD8^+^ T cells secreting cytokines of IFN-γ, IL-2, TNF-α, or IL-17 in the spleen of mice in Ad-AE-R2 group and BCG group in response to stimulations with AE, R2, AE + R2, or BCG WCL proteins in vitro. (**A**) In Ad-AE-R2 immunized mice (n = 6); (**B**) in BCG immunized mice (n = 6).

Furthermore, we analyzed the cytokine profiles of CD4^+^ and CD8^+^ T cells in the spleen of mice following stimulation with AE, R2, AE + R2, and BCG WCL proteins. Interestingly, similar cytokine profiles were observed between the Ad-AE-R2 group and the BCG group for CD4^+^ and CD8^+^ T cells. Among CD4^+^ T cells, IL-2-producing CD4^+^ T cells constituted the majority, followed by fractions of IFN-γ, TNF-α, and IL-17 single cytokine-producing CD4^+^ T cells. There was no statistical difference in the total percentage of cytokine-producing CD4^+^ T cells between Ad-AE-R2 and BCG groups (Figure 7). For CD8^+^ T cells, IFN-γ-producing CD8^+^ T cells were the predominant cytokine-producing CD8^+^ T cells, followed by TNF-α and IL-17 single cytokine-producing CD8^+^ T cells in both groups. Similarly, no statistical difference existed in the total percentage of cytokine-producing CD8^+^ T cells between the Ad-AE-R2 and BCG groups (Figure 8).

### 4.5. Intranasal Immunization with Ad-AE-R2 Could Rapidly Induce Th1-Biased Humoral Immune Responses

**Figure 9:**
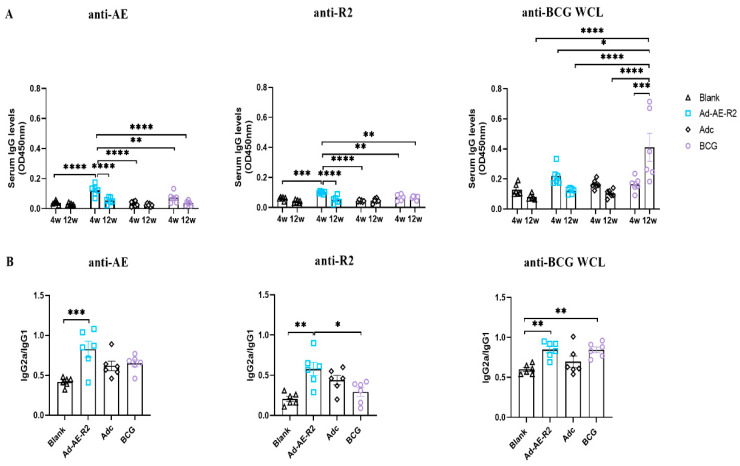
Humoral immune responses in sera of mice (n = 6 per group). (**A**) Antigen-specific IgG levels at 4 and 12 weeks after immunization. (**B**) The IgG2a/IgG1 ratios of antigen-specific IgGs at 12 weeks after immunization. *, *p* < 0.05; **, *p* < 0.01; ***, *p* < 0.001; ****, *p* < 0.0001.

Humoral immunity has also been shown to play a protective role in Mtb infections [28,29]. Therefore, we investigated whether intranasal immunization with Ad-AE-R2 could elicit humoral immune responses in immunized mice by measuring antigen-specific IgG levels in sera using an ELISA test. The results demonstrated that both anti-AE and anti-R2 IgG levels in the sera of mice in the Ad-AE-R2 group were significantly increased at 4 weeks post immunization. However, these antibody levels declined at 12 weeks post immunization and reached similar levels as those in the blank group. In contrast, compared to the blank group, there were no changes in the levels of anti-AE, R2, and BCG WCL IgG antibodies in the BCG group at 4 weeks after immunization. However, the level of anti-BCG WCL IgG increased significantly at 12 weeks after immunization and was higher than that in other groups both at 4 and 12 weeks after immunization (Figure 9A). These findings suggest that intranasal immunization with Ad-AE-R2 could induce antigen-specific humoral immune responses in the sera of mice. Furthermore, it is worth noting that intranasal immunization with Ad-AE-R2 induces rapid humoral immune responses compared to BCG-induced responses, which are relatively slow.

We also quantified the ratios of IgG2a/IgG1 to elucidate systemic Th1/Th2-type responses. The results showed that all three ratios for anti-AE, R2 and BCG WCL IgGs were higher in the Ad-AE-R2 group compared to those in the blank group. Conversely, only the ratio of anti-BCG WCL IgG in the BCG group was higher than that in the blank group. Nevertheless, our results indicate that both intranasal immunization with Ad-AE-R2 and subcutaneous immunization with BCG induce a Th1-biased humoral immunity (Figure 9B).

### 4.6. Intranasal Immunization with Ad-AE-R2 Could Confer Superior Protection in the Lung of Mice Compared to BCG

**Figure 10:**
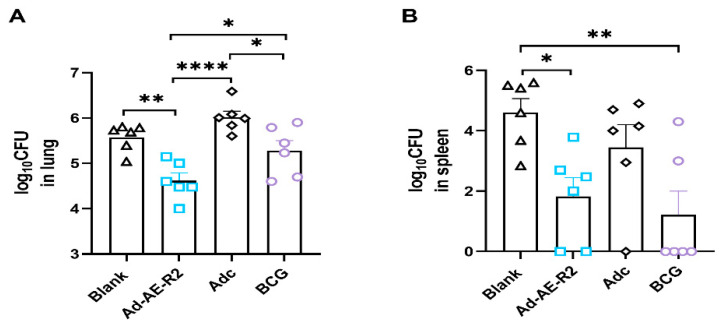
The bacteria burden in the lungs and spleens of mice (n = 6 per group). (**A**) Bacteria numbers in the lung. (**B**) Bacteria numbers in the spleen. *, *p* < 0.05; **, *p* < 0.01; ****, *p* < 0.0001.

After 12 weeks of immunization, the immune protection in mice was assessed through intratracheal challenge with a high-dose BCG-Beijing strain. The bacterial burden was evaluated 4 weeks after the challenge. The results demonstrated that intranasal immunization with Ad-AE-R2 significantly reduced the bacterial burden in the lungs of immunized mice, resulting in a 1.40 log reduction compared to the blank group, and a 0.66 log10 reduction compared to the BCG group. In contrast, there was no significant difference in bacterial burden in the lung of mice between the BCG group and the blank group, indicating that subcutaneous immunization with BCG did not provide effective immune protection against lung infection (Figure 10A). Our results suggest that intranasal immunization with Ad-AE-R2 could provide better immune protection in the lungs of mice than subcutaneous immunization with BCG.

Regarding spleen infection, both intranasal immunization with Ad-AE-R2 and subcutaneous immunization with BCG led to substantial reductions of 2.78 log10 and 3.39 log10, respectively, when compared to the blank group. However, there was no statistical difference in the bacteria burden in the spleen of mice between Ad-AE-R2 and BCG groups, suggesting that both vaccinations induced similar immune protection in the spleen of mice (Figure 10B).

## 5. Discussion

Over the past few decades, significant efforts have been devoted to the development of novel TB vaccines, with some leading candidates progressing through various stages of clinical evaluation. However, there is still no vaccine available to replace or boost BCG. Mtb bacilli have a complex life cycle within the host and express different antigens during both acute and latent stages of infection [9]. Incorporating multi-stage antigens has emerged as an important strategy in the development of novel TB vaccines [10,11,12,13]. Furthermore, the vaccination route plays a crucial role in determining the quality and localization of the resultant immune responses [30,31]. Respiratory mucosal immunity plays a key role in protective immunity against Mtb infection [7,18,22]. Eliciting Mtb-specific immune responses at mucosal sites, such as the respiratory tract, may provide enhanced protection against Mtb infection compared to systemic immunization.

Mucosal immunity at the mucosal surfaces is primarily induced by mucosal immunization through oral, nasal, sublingual routes, etc. [32,33]. SIgA is the predominant antibody isotype at mucosal surfaces, which effectively prevents bacteria adsorption and blocks the entrance of mycobacteria into the lungs [23]. In our study, we found that 12 weeks after immunization, there was no difference in sIgA content in the BALF of mice between the Ad-AE-R2 group and the blank group; however, it significantly increased in the BCG group. Previous studies have demonstrated the successful induction of sIgA in the airway of mice via mucosal immunization when using Ad-vectored vaccines [17,34,35,36]. Additionally, these studies have shown that peak sIgA levels are typically reached around 3 to 4 weeks post immunization followed by a rapid decline [34,35]. Furthermore, some studies have indicated a strong correlation between sIgA levels on mucosal surfaces and serum IgG levels following immunization [34,37]. In our study, we found that the serum antigen-specific IgG level was higher at 4 weeks but declined at 12 weeks after immunization with Ad-AE-R2. Conversely, in the BCG group, the serum antigen-specific IgG level was higher at 12 weeks compared to at 4 weeks post immunization, synchronizing with sIgA levels in BALF of mice. Therefore, we speculated that sIgA level in the BALF of mice in the Ad-AE-R2 group had diminished by week 12 post immunization. Although there was a higher level of sIgA in the BALF of mice in the BCG group at week 12 post immunization, it did not prevent and control subsequent BCG challenge. In fact, prior studies have reported insufficient antibody production following BCG immunization for controlling Mtb infection [38,39,40]. Therefore, considering the important role played by sIgA on mucosal surfaces, targeting and promoting long-term sIgA response should be considered as one strategy of developing improved TB vaccines.

It has been shown that T cells in the respiratory passage are closely associated with protection [41]. Santosuosso M. et al. [42] discovered that administering a recombinant adenovirus-based TB vaccine expressing Mtb Ag85A (AdAg85A) intranasally resulted in higher numbers of antigen-specific CD4^+^ and CD8^+^ T cells in the respiratory passage. These cells were capable of producing IFN-γ and cytolytic activities. Similarly, Darrah PA. et al. [43] observed that CD4^+^ T cell immunity and NK cells in the respiratory passage were linked to protection after intravenous BCG administration. In our study, we observed that intranasal immunization with Ad-AE-R2 decreased the percentage of CD4^+^ T cells but increased the percentage of CD8^+^ T cells, specifically CD8^+^ TRM cells, in the BALF of mice. There were no changes in the percentages of CD4^+^ and CD8^+^ T cell subsets and TRM cells in the BALF of mice in the BCG group. Since mice immunized with Ad-AE-R2 exhibited a lower bacteria burden compared to those in the BCG group while maintaining similar local lung immunity, we propose that CD8^+^ T cells, particularly CD8^+^ TRM cells in the respiratory passage of mice in the Ad-AE-R2 group, may confer immune protection against intratracheal BCG challenge early during infection. Numerous studies have emphasized the crucial role played by CD8^+^ T cells in controlling Mtb infection [44,45,46,47]. Winchell CG. et al. [47] demonstrated that the loss of CD8^+^ innate or adaptive lymphocytes significantly impaired early control over Mtb infection, leading to the increased formation of granulomas, lung inflammation, and bacterial burden. TRM cells act as frontline defenders within adaptive immunity against Mtb infection by rapidly activating and proliferating during the early stages of Mtb infection, which is crucial for protection against initial Mtb infection [24]. Therefore, our study provides further evidence supporting a strong correlation between the presence of T cells in the respiratory passage and protective immune responses. Moreover, we propose that enhancing CD8^+^ T cell responses, particularly CD8^+^ TRM cells within the respiratory passage, holds promise for the development of effective TB vaccines.

The lung is the primary organ invaded by Mtb. Inducing local Mtb-specific immunity in the lung is crucial for controlling Mtb infection [20,25]. In our study, we discovered that intranasal immunization with Ad-AE-R2 and subcutaneous immunization with BCG elicited comparable T cell immune responses in the pulmonary compartment of mice. This included an increase in the percentage of CD8^+^ T cells and the promotion of CD4^+^ and CD8^+^ TRM cell generation within the lung. Furthermore, the cytokine-producing profile of CD4^+^ and CD8^+^ T cells in the lung of mice was also similar between the Ad-AE-R2 group and the BCG group, except for a higher percentage of cytokine-producing CD8^+^ T cells in the Ad-AE-R2 group compared to the BCG group. Upon intratracheal challenge with a high dose of BCG, mice immunized with Ad-AE-R2 exhibited significantly reduced bacterial burden in their lungs compared to those vaccinated with BCG. Therefore, we believe that an increased proportion of cytokine-producing CD8^+^ T cells may confer enhanced protection in the pulmonary compartment, emphasizing once again that targeting CD8^+^ T cells should be considered as a strategy for developing novel TB vaccines.

It has been proposed that local lung immunity may regulate the growth of pathogens in the lungs, while systemic immunity could potentially control the growth of pathogens that escape to other tissues [25]. The spleen functions as a guardian for systemic immunity, initiating and sustaining immune responses against blood-borne pathogens [48]. Our study demonstrates that intranasal immunization with Ad-AE-R2 induces enduring antigen-specific immunity in the spleen of mice, including the antigen-specific proliferation of splenolymphocytes and an increase in the proportions of CD8^+^ T cells and Tcm cells. In comparison, intranasal immunization with Ad-AE-R2 primarily stimulates CD8^+^ T cell immunity, while subcutaneous immunization with BCG predominantly induces CD4^+^ T cell immunity in the spleen of mice. Although there is a disparity between inducing CD4^+^ or CD8^+^ T cell immunity in the spleens of mice, both vaccinations have equal capability to generate Tcm cells. When challenged by a high dose of BCG intratracheally, there was no difference in the bacteria burden in the spleens of mice between the Ad-AE-R2 group and the BCG group. Hence, we think that Tcm cells play a key role in protecting the spleen of mice against the challenge.

One indicator of the initiation of defensive responses would be the capacity of the host immune response to manage or eradicate mycobacteria. In order to carry out experiments on the effectiveness of vaccines, which necessitate Mtb challenge, biosafety level 3 facilities are required. However, these facilities are costly to maintain and often overbooked, which can create a bottleneck when testing vaccine candidates. Therefore, there have been studies assessing the feasibility of utilizing BCG challenge instead. These studies demonstrate that BCG challenge could aid in prioritizing competing TB vaccine candidates, investigating immune responses to mycobacteria, and providing a more cost-effective and efficient approach to testing new vaccine candidates [49,50,51]. In our study, we assessed the effectiveness of the vaccines through a high-dose intratracheal BCG challenge. Based on the results of the challenge, we believe that the protective immunity induced by both vaccinations indicates their effectiveness in protecting against lung and spleen infections in immunized mice. Our study once again supports that BCG challenge is a convenient and efficient method for evaluating vaccine candidate efficacy. However, since BCG cannot enter a dormant state like Mtb during infection, it is still necessary to evaluate the protective effect of Ad-AE-R2 on dormant bacteria using an animal model infected with Mtb.

In summary, our study demonstrates that the intranasal administration of Ad-AE-R2, an Ad vectored TB vaccine expressing multi-stage antigens of Mtb, not only induces long-term mucosal immunity in the airway and lungs but also elicits enduring systemic memory immunity in the spleens of immunized mice. Compared with subcutaneous immunization with BCG, intranasal immunization with Ad-AE-R2 provided superior protection in the lungs of mice while conferring similar protection in the spleens of mice. Additionally, we provide evidence supporting enhancing CD8^+^ T cell immunity as an effective strategy for improving TB vaccines and emphasize the importance of eliciting systemic memory immunity for a successful mucosal TB vaccine. Furthermore, our study demonstrates that BCG challenge serves as a convenient and efficient method to evaluate the effectiveness of vaccine candidates.

## Data Availability

The data presented in this study are available in this article and Supplementary Materials.

## Supplementary Materials

The following supporting information can be downloaded at: https://www.mdpi.com/article/10.3390/vaccines12091022/s1, Figure S1: (A) Diagrammatic representation of the gene cassette of Ag85B-ESAT6 (AE) and Rv2031c-Rv2626c (R2) fusion proteins and the resultant Ad-AE-R2 vector. Ag85B and ESAT6, Rv2031c and Rv2626c were fused by a flexible hydrophobic (Gly3Ser)4 linker. The AE gene cassette was under control of a cytomegalovirus (CMV) promoter, the R2 gene cassette was under control of a human elonggation factor 1α (EF1α) promoter. The drawings are not to scale. LITR & RITR, the left and right terminal repeats. (B) qPCR validation of AE and R2 gene cassettes in HEK293T cells infected with Ad-AE-R2. ****, *p* < 0.001. (C) Expression of AE, R2 fusion proteins in eukaryotic cells for Ad-AE-R2 vector detected by indirect mmunofluorescence assay (IFA) using anti-Ag85B mAb (b), anti-ESAT6 mAb (c), anti-Rv2031c mAb (d), and anti-Rv2626c mAb (e), respectively. Green fluorescence could be detected in macrophages cells infected with Ad-AE-R2 but not with control vector Adc (a); Figure S2: Flow cytometry gating strategy for CD4^+^ and CD8^+^ TRM cells.
