# Supplementary material for: Intranasal Immunization with a Recombinant Adenovirus Encoding Multi-Stage Antigens of Mycobacterium tuberculosis Preferentially Elicited CD8+ T Cell Immunity and Conferred a Superior Protection in the Lungs of Mice than Bacillus Calmette–Guerin
Source: Vaccines (Basel). 2024 Sep 6;12(9):1022. doi: 10.3390/vaccines12091022 (PMC11436211; doi:10.3390/vaccines12091022)
Supplement: Supplementary file 1 [file vaccines-12-01022-s001.zip › vaccines-3104174-supplementary.pdf]

Supplemental Figures and Figure legends

A

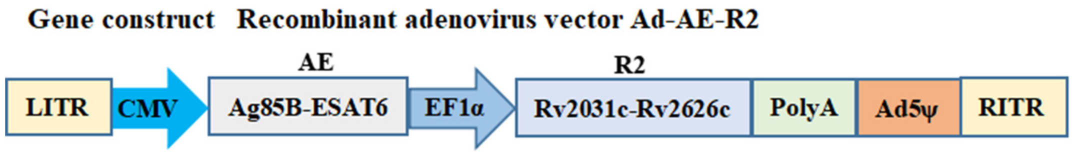

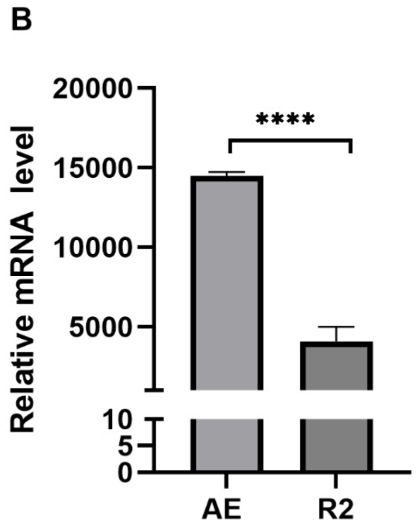

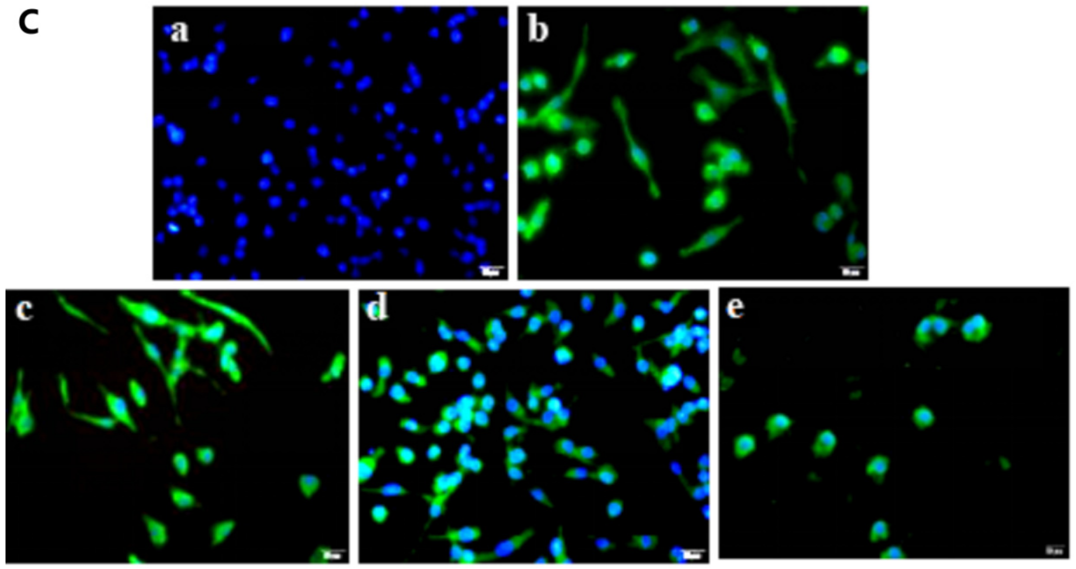

Supplemental Figure S1. (A) Diagrammatic representation of the gene cassette of Ag85B-ESAT6 (AE) and Rv2031c-Rv2626c (R2) fusion proteins and the resultant

Ad-AE-R2 vector. Ag85B and ESAT6, Rv2031c and Rv2626c were fused by a flexible hydrophobic (Gly3Ser)4 linker. The AE gene cassette was under control of a cytomegalovirus (CMV) promoter, the R2 gene cassette was under control of a human elonggation factor 1α (EF1α) promoter. The drawings are not to scale. LITR & RITR, the left and right terminal repeats. (B) qPCR validation of AE and R2 gene cassettes in HEK293T cells infected with Ad-AE-R2. ****, $p < 0.001$. (C) Expression of AE, R2 fusion proteins in eukaryotic cells for Ad-AE-R2 vector detected by indirect mmunofluorescence assay (IFA) using anti-Ag85B mAb (b), anti-ESAT6 mAb (c), anti-Rv2031c mAb (d), and anti-Rv2626c mAb (e), respectively. Green fluorescence could be detected in macrophages cells infected with Ad-AE-R2 but not with control vector Adc (a).

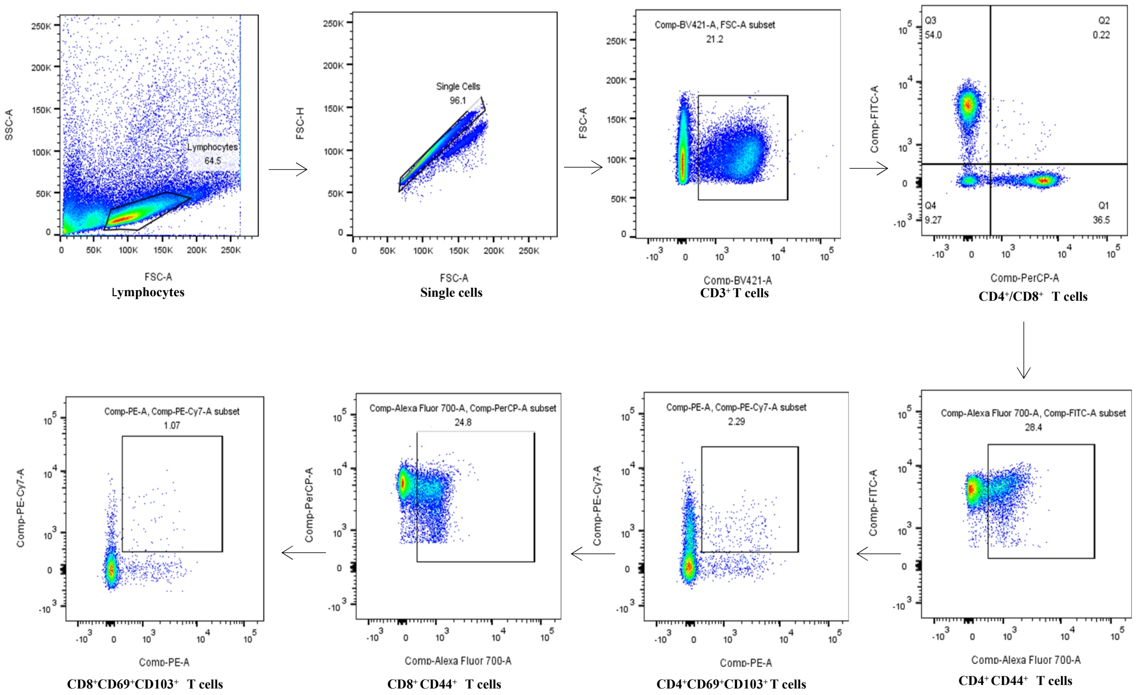

Supplemental Figure S2. Flow cytometry gating strategy for $CD4^+$ and $CD8^+$ TRM cells.
